# Fast semi-automated lesion demarcation in stroke

**DOI:** 10.1016/j.nicl.2015.06.013

**Published:** 2015-07-17

**Authors:** Bianca de Haan, Philipp Clas, Hendrik Juenger, Marko Wilke, Hans-Otto Karnath

**Affiliations:** a Center of Neurology, Division of Neuropsychology, Hertie-Institute for Clinical Brain Research, University of Tübingen, Tübingen, Germany; b Department of Pediatric Neurology and Developmental Medicine, Children's Hospital, University of Tübingen, Germany; c Experimental Pediatric Neuroimaging, Department of Neuroradiology, Children's Hospital, University of Tübingen, Germany; d Department of Pediatrics, Klinikum Rechts Der Isar, Technical University of München, Germany; e Department of Psychology, University of South Carolina, Columbia, SC, USA

**Keywords:** Stroke, Lesion segmentation, Semi-automated, Lesion–behaviour mapping analyses, SPM toolbox

## Abstract

Lesion–behaviour mapping analyses require the demarcation of the brain lesion on each (usually transverse) slice of the individual stroke patient's brain image. To date, this is generally thought to be most precise when done manually, which is, however, both time-consuming and potentially observer-dependent. Fully automated lesion demarcation methods have been developed to address these issues, but these are often not practicable in acute stroke research where for each patient only a single image modality is available and the available image modality differs over patients. In the current study, we evaluated a semi-automated lesion demarcation approach, the so-called Clusterize algorithm, in acute stroke patients scanned in a range of common image modalities. Our results suggest that, compared to the standard of manual lesion demarcation, the semi-automated Clusterize algorithm is capable of significantly speeding up lesion demarcation in the most commonly used image modalities, without loss of either lesion demarcation precision or lesion demarcation reproducibility. For the three investigated acute datasets (CT, DWI, T2FLAIR), containing a total of 44 patient images obtained in a regular clinical setting at patient admission, the reduction in processing time was on average 17.8 min per patient and this advantage increased with increasing lesion volume (up to 60 min per patient for the largest lesion volumes in our datasets). Additionally, our results suggest that performance of the Clusterize algorithm in a chronic dataset with 11 T1 images was comparable to its performance in the acute datasets. We thus advocate the use of the Clusterize algorithm, integrated into a simple, freely available SPM toolbox, for the precise, reliable and fast preparation of imaging data for lesion–behaviour mapping analyses.

## Highlights

• We evaluated a semi-automated lesion demarcation approach in stroke patients.
• We compared our approach to the gold standard of manual lesion demarcation.
• We investigated typically used image modalities in acute clinical settings.
• Precision and reliability of our approach were comparable to the gold standard.
• Our approach was significantly faster than the gold standard.

## 1 Introduction

Lesion–behaviour mapping analyses that enable the mapping of clinical deficits to the location of brain damage represent an indispensable method for the study of human brain function (Rorden and Karnath, 2004). Specifically, their ability to demonstrate a causal relationship between a structural lesion and an observable deficit, means that they are an important addition to modern neuro-imaging techniques that allow us to measure and localise the neural correlates of task performance in healthy subjects. Lesion–behaviour mapping analyses are most commonly performed in stroke patients as stroke lesions are focal, well-defined and clearly visible in patients' brain images (as opposed to e.g., tumour lesions where the full extent of affected brain tissue often extends beyond the visible tumour borders (see e.g. Karnath and Steinbach, 2011). Lesion–behaviour mapping analyses require the demarcation of the lesion border on each, usually transverse, slice of the individual patient's brain image. To date, this is generally thought to be most precise when done manually, which is, however, both time-consuming and potentially observer-dependent (Ashton et al., 2003). As a consequence, sample sizes studied in lesion–behaviour mapping studies are typically small, which severely limits statistical power and prohibits the application of new innovative methods like multivariate pattern analysis (MVPA) to lesion data (shown in e.g. Smith et al., 2013 to be possible in sufficiently large datasets).

To address these disadvantages associated with manual lesion demarcation, several fully automated lesion demarcation methods have been proposed over the years. As the name implies, these methods do not require any user interaction and as such are both less time-consuming and, theoretically, less observer-dependent than manual lesion demarcation. The most common type of fully automated lesion demarcation methods rely on the comparison of each patient brain to a reference dataset of neurologically healthy control brains (Gillebert et al., 2014; Mah et al., 2014; Seghier et al., 2008; Stamatakis and Tyler, 2005; Wilke et al., 2003). In this method, the brain images of both the patient and the controls are normalised to the same template. Subsequently, the patient brain is statistically compared to the control dataset on a voxel-by-voxel basis to identify “atypical areas” (i.e. suspected lesions) where image intensity differs between patient and controls.

The disadvantage of these fully automated lesion demarcation methods, however, is that they may be less precise than manual demarcation as, in contrast to a human observer, they may fail to identify imaging artefacts. Moreover, a large practical drawback is that these fully automated lesion demarcation methods require the collection of a sufficiently large control dataset (Wilke et al., 2014). This drawback is compounded by the fact that these fully automated lesion demarcation methods can only be applied to stroke patient data that have the same image modality as the control dataset. These issues are particularly problematic when performing lesion–behaviour mapping analyses with acute stroke patients, where scientists typically have to rely on imaging data obtained in a regular clinical setting. In most neurological departments, image collection protocols performed at admission follow clinical, not scientific criteria. For example, computed tomography (CT) remains the modality of choice for many stroke patients at admission (El-Koussy et al., 2014), with advantages typically including the definitive detection of haemorrhage, speed and cost-efficiency, as well as fewer exclusion criteria than magnetic resonance (MR) imaging. Due to the recent development of CT templates for spatial normalisation (Rorden et al., 2012), these images no longer have to be excluded from lesion–behaviour mapping analyses. However, having to rely on clinical CT and MR images has several consequences for anatomo-behavioural studies in acute stroke: First, often only a single image modality is available per patient. Second, the available image modality often differs between patients (depending on time since stroke, contraindications for MRI, etc.) and thirdly, even within an image modality, image acquisition parameters may differ between patients. This limits the practical applicability of the fully automated lesion demarcation methods described above in acute patient settings.

A possible solution to these issues has recently been offered in the context of a semi-automated lesion demarcation approach developed by Clas et al. (2012), the so-called Clusterize algorithm (“*Clusterize*”). Semi-automated lesion demarcation methods typically combine a fully automated detection of “abnormalities” (i.e. potential lesions) with manual editing by the user to determine the final location and extent of the lesion (e.g. Wilke et al., 2011). In doing so, these methods aim to combine the best of both worlds: the presence of fully automated steps makes these methods less time-consuming than manual lesion demarcation, while mandatory user interaction results in lesion demarcation that is more precise and/or less error-prone (in the sense that they are closer to the results from the current gold standard, manual demarcation) than that obtained by fully automated lesion demarcation methods. *Clusterize* combines a fully-automated clustering of the image (using automatically-defined local intensity maxima and iterative region growing) with a manual, interactive selection and modification of the clusters-of-interest. Interestingly, while originally developed to determine demyelination load in metachromatic leukodystrophy using T2 images, *Clusterize* should theoretically be able to demarcate lesions regardless of image modality, as long as the lesion can be separated from directly adjacent healthy tissue on the basis of image intensity (note, however, that this is also a mandatory prerequisite when performing manual lesion delineation). Thus, this algorithm might provide a practical, fast, and easy to implement option to delineate the lesions of acute stroke patients.

In the current study, we therefore aimed to evaluate the performance of *Clusterize* in acute stroke patients scanned with a range of common image modalities (CT, DWI, T2FLAIR). We compared the results of *Clusterize* to the results obtained with manual lesion demarcation, with respect to the final lesion map, processing time, and inter-rater reliability.

## 2 Methods

### 2.1 Imaging data

Three ischaemic acute stroke datasets were used, with a total of *n* = 44 images (see Table 1 for patient characteristics of each of the 3 acute datasets). The first dataset contained 13 CT images from patients with acute unilateral stroke, covering the whole brain with an in-plane resolution of 0.4 × 0.4 mm and a slice thickness varying between 4.5 and 5.1 mm. The second dataset contained 16 diffusion-weighted MR images (DWI) from patients with acute unilateral stroke, covering the whole brain with an in-plane resolution of 0.9 × 0.9 mm and a slice thickness varying between 4.8 and 6.3 mm. The third dataset contained 15 T2FLAIR-weighted MR images from patients with acute unilateral stroke covering the whole brain. Nine of these images had an in-plane resolution of 0.9 × 0.9 mm and a slice thickness varying between 4.4 and 8.0 mm, the remaining 6 images had an in-plane resolution of 1.0 × 1.0 mm and a slice thickness varying between 2.0 and 2.2 mm. These images from acute ischaemic stroke patients were collected as part of the routine clinical investigation after the patient was admitted to the Tübingen Center of Neurology due to acute onset of neurological symptoms.

### 2.2 Manual lesion demarcation approach

For each patient image of each of the 3 datasets, manual demarcation of each brain lesion was performed by a trained rater. For each patient, the boundary of the lesion was delineated directly on the individual brain image for every single transverse slice using MRIcroN software (http://www.mricro.com/mricron). The time required for manual lesion demarcation of each image of each of the 3 datasets was recorded in minutes with a stopwatch.

### 2.3 Semi-automated lesion demarcation approach with the Clusterize algorithm

For each patient image of each of the 3 datasets, semi-automated demarcation of each brain lesion was also performed with *Clusterize* (Clas et al., 2012). This algorithm has been integrated into an easy-to-use, freely available SPM toolbox (http://www.medizin.uni-tuebingen.de/kinder/en/research/neuroimaging/software/). We used this toolbox with SPM8, running under Matlab R2013b (The Mathworks, Inc., Natick, MA).

First, we used *Clusterize* to perform a fully automated clustering of the image on the basis of local intensity maxima and iterative region growing (see Clas et al. (2012) for full details). To avoid oversegmented results, a cluster extend volume threshold of 150 mm^3^ (i.e. 0.15 cm^3^) was used for the initiation of a cluster. To eliminate non-contributing background voxels, a lower intensity threshold of 20% was used and the intensity threshold was subsequently iteratively adapted in steps of 1%. For each image slice, this ultimately results in a 3-dimensional matrix with one 2-dimensional plane for each intensity threshold containing the clusters found at that intensity threshold. The fully automated clustering was run in overnight batch jobs. Unattended processing time was approximately 30 min for each image in the acute CT dataset and approximately 1–2 min for each image in the acute DWI and acute T2FLAIR datasets.

After this fully automated clustering of the image, the same trained rater that also performed the manual lesion demarcation used *Clusterize* to interactively select the cluster(s) corresponding to the lesion(s) (again see Clas et al. (2012) for full details). If necessary, the size of the cluster(s) was/were adapted by modifying the intensity threshold (i.e. the optimal intensity plane was interactively selected from the automatically computed 3-dimensional matrix). The time required for this manual interactive selection and adaptation of the cluster(s)-of-interest of each image of each of the 3 datasets was again recorded in minutes with a stopwatch.

### 2.4 Comparison between manual and semi-automated lesion demarcation approach

For each image of each dataset, the trained rater performed the manual lesion demarcation first, followed by the semi-automated lesion demarcation. Thus, the comparison between the manual and semi-automated lesion demarcation approaches was potentially confounded by effects of familiarity with the patient image. To address this confound, we had the same trained rater redo the manual and semi-automated lesion demarcation of all 3 datasets. Subsequently, when comparing the manual and the semi-automated lesion demarcation approaches, we used the data from these second demarcation instances where the rater was familiar with the patient image during both manual and semi-automated lesion demarcation.

To compare the lesion maps generated by *Clusterize* to the lesion maps generated by the gold standard manual delineation, we calculated Dice's similarity index (DSI; Dice, 1945) for each image for each of the 3 datasets, using the following formula:

$$DSI_{manual,semi-automated}=\frac{\text{2}(manual\wedge semi-automated)}{(manual+semi-automated).}$$

The DSI is thus calculated by dividing twice the overlap between the two maps by their sum, thereby taking into account both false positives and false negatives, and ranges from 0 (no agreement) to 1 (perfect agreement). The DSI has been compared with the kappa statistic (Zou et al., 2004) and accordingly DSI values between .6 and .8 have been considered good and DSI values >.8 have been considered near perfect (Landis and Koch, 1977).

As the DSI can be insensitive to differences between the maps in situations where the overlap between the maps is high (DSI > .8), we additionally calculated Jaccard's coefficient of community (JCC; Jaccard, 1912), which is more sensitive to differences between the maps in these situations, using the following formula:

$$JCC_{manual,semi-automated}=\frac{\text{2}(manual\wedge semi-automated)}{(manual\vee semi-automated).}$$

The JCC is thus calculated by dividing twice the overlap between the two maps by their union and also ranges from 0 to 1. The relationship between the DSI and the JCC is expressed as JCC = DSI / (2 − DSI). Consequently, JCC values >.54 can be considered good and JCC values >.67 can be considered near perfect.

To assess whether the average DSI and/or JCC differed between the 3 acute datasets, we performed Kruskal–Wallis tests. To compare the time required between manual and semi-automated lesion demarcation, we performed a Wilcoxon signed-rank test for each of the 3 acute datasets. Finally, to assess whether either DSI values, JCC values or differences in processing time required between manual and semi-automated lesion demarcation were associated with lesion volume, we performed Spearman's rank correlation analyses. A Bonferroni correction for multiple comparisons was applied when appropriate and significance was assumed when *p* < .05.

### 2.5 Inter-rater reliability

To assess inter-rater reliability, a second trained rater independently redid the manual and semi-automated lesion demarcation of all 3 datasets. We used the intraclass correlation coefficient (ICC [model 2,1]) to quantify the inter-rater reliability of the volumes of the lesion maps, both for the manual and the semi-automated lesion demarcation approach for each of the 3 acute datasets. To assess whether the volumes of the lesion maps differed systematically between the raters, the lesion volumes from the first and second rater for each of the 3 datasets were statistically compared with Mann–Whitney-*U* tests. Again, a Bonferroni correction for multiple comparisons was applied when appropriate and significance was assumed when *p* < .05.

### 2.6 Confirmation of performance in chronic stroke patients

As mentioned in the Introduction section, performance of *Clusterize* should theoretically be independent of image modality. As such, it should be able to demarcate lesions not only in acute stroke patients with CT, DWI or T2FLAIR images, but also in chronic stroke patient where the typical image modality is T1. To confirm this, we additionally assessed performance of *Clusterize* in a dataset containing T1 images from patients with chronic unilateral stroke. This dataset contained 11 T1-weighted MRI images from patients with chronic perinatally acquired unilateral medial cerebral artery stroke (5 male, 6 female; mean age 15.5 years, age range 10–30; 7 left hemispheric stroke, 4 right hemispheric stroke). These images were collected as part of a study on reorganisation following early brain lesions (Juenger et al., 2011) and covered the whole brain with a resolution of 1.0 × 1.0 × 1.0 mm. For this dataset, manual and semi-automated lesion demarcation were performed as described above and by the same trained raters, with the only exception that the first rater performed the manual and semi-automated lesion demarcation only once. Thus, in this dataset the comparisons between manual and semi-automated lesion demarcation might be confounded by effects of familiarity with the patient image.

## 3 Results

**Fig. 1:**
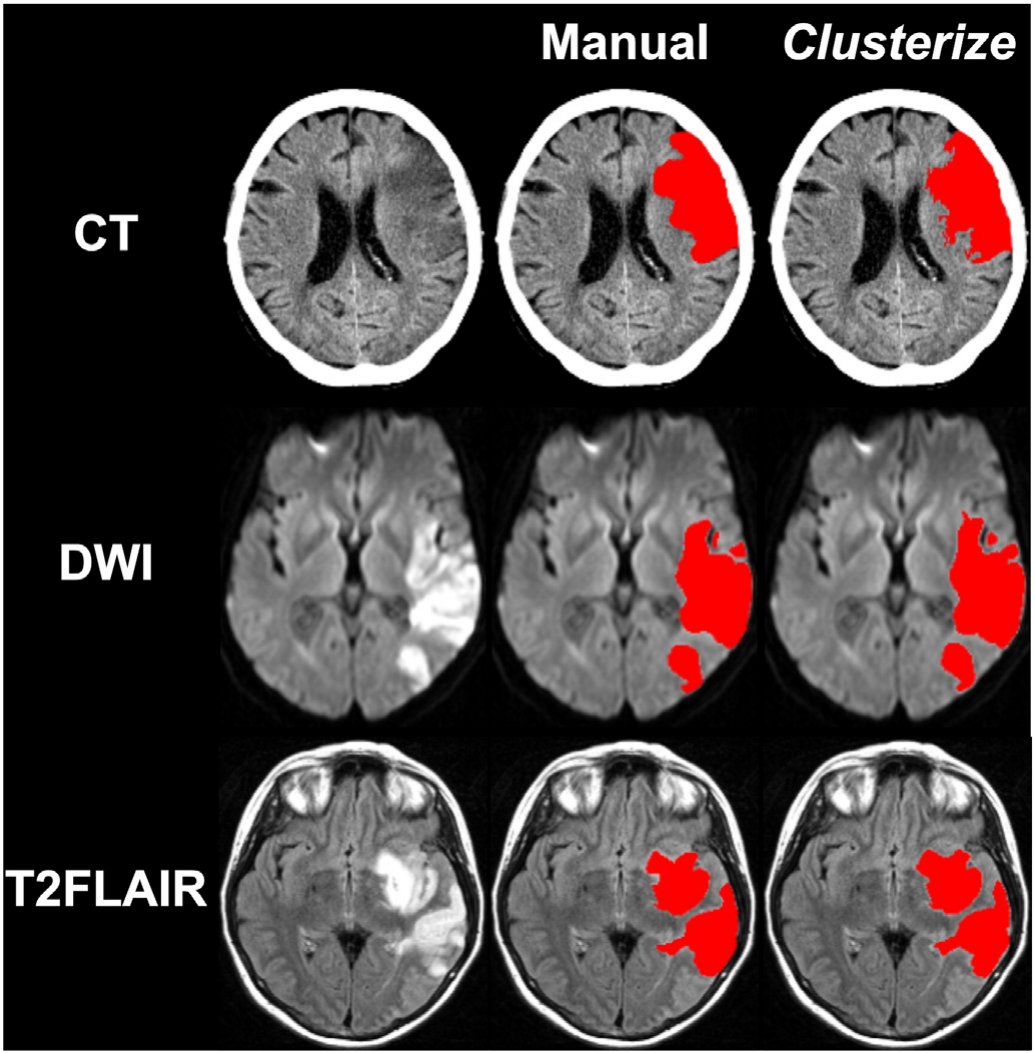
An example of the results from both manual and semi-automated lesion demarcation with *Clusterize* in a single patient for each of the 3 acute datasets.

An example of the results from both manual and semi-automated lesion demarcation with *Clusterize* for a single patient of each of the 3 acute datasets is shown in Fig. 1.

### 3.1 Manual lesion demarcation approach

For the acute CT dataset, the average lesion volume was 28.64 (standard deviation [SD] = 33.81, 95% confidence interval [CI] =  ±18.38) cm^3^ for the first rater and 31.24 (SD = 36.31, CI = ±19.73) cm^3^ for the second rater; this difference was not significant (U_26_ = .385, *p* > .999) and the ICC was .993 (CI = .976 to .998). For the acute DWI dataset, the average lesion volume was 22.03 (SD = 31.09, CI = ±15.23) cm^3^ for the first rater and 22.51 (SD = 33.42, CI = ±16.37) cm^3^ for the second rater; this difference was not significant (U_32_ < .188, *p* > .999) and the ICC was .973 (CI = .925 to .990). For the acute T2FLAIR dataset, the average lesion volume was 15.59 (SD = 19.29, CI = ±9.76) cm^3^ for the first rater and 20.44 (SD = 26.89, CI = ±13.61) cm^3^ for the second rater; this differences was once again not significant (U_30_ = .394, *p* > .999) and the ICC was .933 (CI = .813 to .977).

Manual lesion demarcation required on average 19.6 (SD = 16.2, CI = ± 8.8) minutes per patient for the acute CT dataset, 18.4 (SD = 21.4, CI = ±10.5) minutes per patient for the acute DWI dataset and 25.1 (SD = 28.1, CI = ±14.2) minutes per patient for the acute T2FLAIR dataset.

### 3.2 Semi-automated lesion demarcation approach with the Clusterize algorithm

For the acute CT dataset, the average lesion volume was 28.24 (SD = 33.16, CI = ±18.02) cm^3^ for the first rater and 27.54 (SD = 32.86, CI = ±17.86) cm^3^ for the second rater; this difference was not significant (U_26_ = .026, *p* > .999) and the ICC was .994 (CI = .980 to .998). For the acute DWI dataset, the average lesion volume was 21.71 (SD = 31.45, CI = ±15.41) cm^3^ for the first rater and 19.39 (SD = 28.51, CI = ±13.97) cm^3^ for the second rater; this difference was not significant (U_32_ = .490, *p* > .999) and the ICC was .962 (CI = .895 to .987). For the acute T2FLAIR dataset, the average lesion volume was 15.59 (SD = 19.54, CI = ±9.89) cm^3^ for the first rater and 17.95 (SD = 21.96, CI = ±11.11) cm^3^ for the second rater; this difference was once again not significant (U_30_ = .270, *p* > .999) and the ICC was .984 (CI = .953 to .995).

Semi-automated lesion demarcation took on average 4.4 (SD = 3.50, CI = ±1.9) minutes per patient for the acute CT dataset, 2.3 (SD = 1.7. CI = ±0.8) minutes per patient for the acute DWI dataset and 3.1 (SD = 2.7, CI = ±1.4) minutes per patient for the acute T2FLAIR dataset.

### 3.3 Comparison between manual and semi-automated lesion demarcation approach

**Fig. 2:**
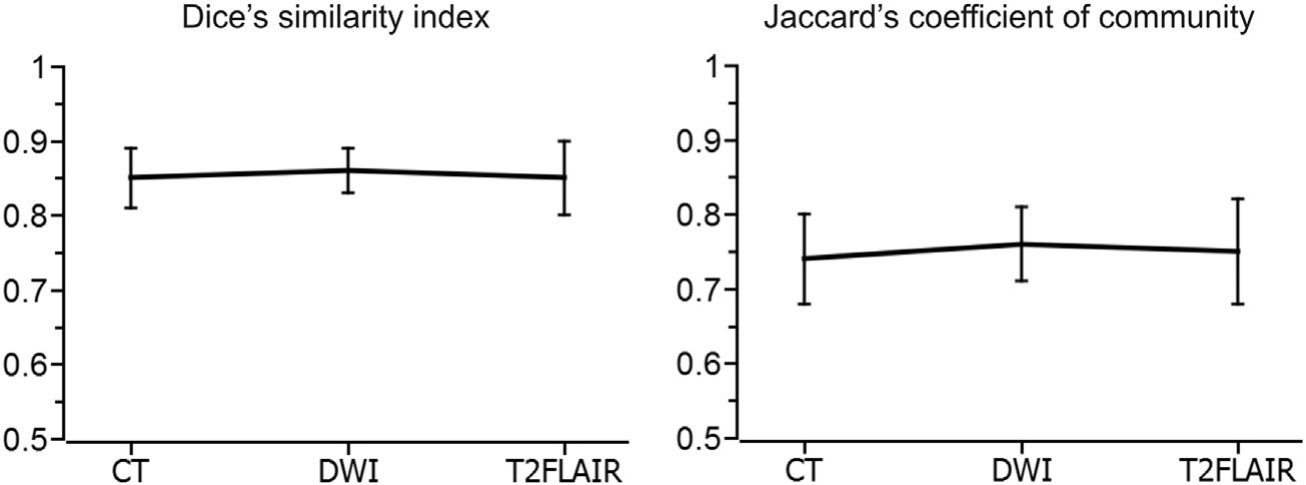
Similarity between the lesion maps from manual and semi-automated lesion demarcation for each of the 3 acute datasets, calculated using either Dice's similarity index (left graph) or Jaccard's coefficient of community (right graph). Error bars denote the 95% confidence interval of the mean.

The average DSI between the manually and the semi-automatically demarcated lesion maps was .85 (SD = .08, CI = ±.04) for the acute CT dataset, .86 (SD = .07, CI = ±.03) for the acute DWI dataset and .85 (SD = .10, CI = ±.05) for the acute T2FLAIR dataset (see Fig. 2) and did not differ significantly between the 3 datasets (H_2_ = .180, *p* = .914). For each of the 3 datasets, DSI values were significantly correlated with lesion volume (CT dataset: ρ_13_ = .742, *p* = .012; DWI dataset: ρ_16_ = .818, *p* < .001; T2FLAIR dataset: ρ_15_ = .696, *p* = .012), with DSI values increasing as lesion volume increased. The average JCC between the manually and the semi-automatically demarcated lesion maps was .74 (SD = .10, CI = ±.06) for the acute CT dataset, .76 (SD = .11, CI = ±.05) for the acute DWI dataset and .75 (SD = .14, CI = ±.07) for the acute T2FLAIR dataset (see Fig. 2) and likewise did not differ significantly between the 3 datasets (H_2_ = .180, *p* = .914). For each of the 3 datasets, JCC values were significantly correlated with lesion volume (CT dataset: ρ_13_ = .742, *p* = .012; DWI dataset: ρ_16_ = .818, *p* < .001; T2FLAIR dataset: ρ_r15_ = .696, *p* = .012), with JCC values increasing as lesion volume increased.

**Fig. 3:**
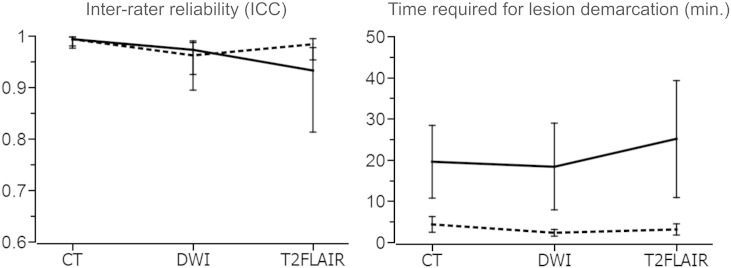
Inter-rater reliability (left graph) and time required for lesion demarcation of lesion volumes (right graph) for both manual (solid lines) and semi-automated (dashed lines) lesion demarcation for each of the 3 acute datasets. Error bars denote the 95% confidence interval of the mean.

For each of the 3 datasets, the inter-rater reliability was comparable between manual and semi-automated lesion demarcation (CT dataset: ICC = .993 for manual vs. .994 for semi-automated; DWI dataset: ICC = .973 for manual vs. .962 for semi-automated; T2FLAIR dataset: ICC = .933 for manual vs. .984 for semi-automated) (see Fig. 3). Moreover, for each of 3 acute datasets, semi-automated lesion demarcation was significantly faster than manual lesion demarcation (CT dataset: W_13_ = 3.185, *p* = .003; DWI dataset: W_16_ = 3.186, *p* = .003; T2FLAIR dataset: Wt_15_ = 3.300, *p* = .003) (see Fig. 3). For each of the 3 datasets, the difference in processing time between manual and semi-automated lesion demarcation was significantly positively correlated with lesion volume (CT dataset: ρ_13_ = .827, *p* < .001; DWI dataset: ρ_16_ = .907, *p* < .001; T2FLAIR dataset: ρ_15_ = .888, *p* < .001). In other words, as lesion volume increased, the reduction in processing time offered by the semi-automated approach became more pronounced.

### 3.4 Confirmation of performance in chronic stroke patients

In the chronic stroke dataset, manual lesion demarcation took on average 140.5 (SD = 80.0, CI = ±47.3) minutes per patient. The average lesion volume was 37.75 (SD = 37.87, CI = ±22.38) cm^3^ for the first rater and 40.18 (SD = 38.85, CI = ±22.96) cm^3^ for the second rater; this difference was not significant (U_22_ = .164, *p* > .999) and the ICC was .995 (CI = .982 to .999). Semi-automated lesion demarcation took on average 32.5 (SD = 19.6, CI = ±11.6) minutes per patient. The average lesion volume was 41.36 (SD = 39.05, CI = ±23.08) cm^3^ for the first rater and 41.41 (SD = 39.04, CI = ±23.07) cm^3^ for the second rater; this difference was once again not significant (U_22_ = .098, *p* > .999) and the ICC was .994 (CI = .979 to .998).

Comparing manual and semi-automated lesion demarcation in chronic stroke patients, produced results highly similar to those obtained in the acute stroke datasets: The average DSI between the manually and the semi-automatically demarcated lesion maps was .87 (CI = ±.04) and the average JCC between the manually and the semi-automatically demarcated lesion maps was .77 (CI = ±.07). As for the acute datasets, both DSI and JCC values in the chronic dataset were significantly positively correlated with lesion volume (DSI: ρr_11_ = .791, *p* = .004; JCC: ρ_11_ = .791, *p* = .004). Moreover, the inter-rater reliability was comparable between manual and semi-automated lesion demarcation approaches (ICC = .995 for manual vs. .994 for semi-automated). Finally, semi-automated lesion demarcation was significantly faster than manual lesion demarcation (W_11_ = 2.934, *p* = .003) and the reduction in processing time achieved by the semi-automated approach became more pronounced with increasing lesion volume (ρ_11_ = .955, *p* < .001).

## 4 Discussion

In the current study, we evaluated the performance of *Clusterize* (Clas et al., 2012) for semi-automated lesion demarcation in acute stroke patients scanned in a range of commonly used image modalities (CT, DWI, T2FLAIR). Our DSI (>.8 in all 3 acute datasets) and JCC (>.7 in all 3 acute datasets) values indicate that the agreement between the lesion maps generated by *Clusterize* and the lesion maps generated manually was excellent, suggesting that the precision of the lesion demarcation of *Clusterize* was comparable to the precision of the current gold standard of manual lesion demarcation. Moreover, the average DSI value in our acute T2FLAIR dataset containing images from stroke patients was comparable to the average DSI value in the T2FLAIR dataset from Clas et al. (2012), who used *Clusterize* to demarcate lesions in metachromatic leukodystrophy patients (.85 in our dataset versus .79 in the dataset from Clas et al.). This suggests that *Clusterize* is a robust tool for semi-automated lesion demarcation regardless of lesion aetiology. Previous studies using fully automated lesion demarcation reported DSIs of .7342 for DWI images (Mah et al., 2014) and around .6 for CT images (Gillebert et al., 2014). Thus, in terms of lesion demarcation precision, the semi-automated Clusterize algorithm outperforms these fully automated lesion demarcation methods i.e. the results obtained with *Clusterize* are closer to the results of manual lesion demarcation than the results obtained with previously suggested fully automated lesion demarcation methods.

Secondly, for all 3 acute datasets, the inter-rater reliability of *Clusterize* was comparable to that obtained after manual lesion demarcation, indicating that the reproducibility of both methods was comparable in all 3 acute datasets. Overall, both raters agreed extremely well for the CT, DWI and T2FLAIR datasets (ICC > .93 for both manual and semi-automated lesion demarcation). Moreover, the inter-rater reliability of Clusterize in our T2FLAIR stroke patient dataset was comparable to the inter-rater reliability of Clusterize in the T2FLAIR metachromatic leukodystrophy patient dataset of Clas et al. (2012) (.984 in our dataset versus .982 in the dataset from Clas et al.), again suggesting that *Clusterize* is robust against differences in lesion aetiology.

Thirdly, our results demonstrate that semi-automated lesion demarcation with *Clusterize* was significantly faster than manual demarcation for all 3 acute datasets with a reduction in processing time of on average 17.8 minutes per patient. For a large-scale analysis (e.g. MVPA) with 100–200 acute stroke patient images, this would amount to approximately 30–60 hours of working time saved. Importantly, this reduction in processing time per patient increased as lesion volume increased: for lesions smaller than 5 cm^3^, the reduction in processing time per patient was only approximately 2 minutes, but this increased to a reduction in processing time per patient of approximately 20 minutes for lesions of 5–30 cm^3^ and reached a maximum reduction in processing time per patient of up to 60 minutes for the largest lesions in our acute datasets with volumes of around 100 cm^3^.

Finally, the performance of *Clusterize* in a chronic T1 dataset was comparable to the performance of *Clusterize* in the acute datasets. As for the acute datasets, the DSI (.87) and JCC (.77) values for the chronic T1 dataset indicated that the agreement between the lesion maps generated by the Clusterize algorithm and the lesion maps generated manually was excellent. These DSI values also again compare favourably to the results from a previous study, where average DSI values of .64 were obtained for T1 images when using a fully automated lesion demarcation method (Seghier et al., 2008). Moreover, the inter-rater reliability of *Clusterize* was comparable to that obtained after manual lesion demarcation (ICC = .995 for manual and .994 for semi-automated lesion demarcation). While the timing measurements cannot be as easily interpreted in this chronic T1 dataset due to potentially confounding effects of the rater's familiarity with the patient images, our results do suggests that the reduction in processing time offered by *Clusterize* over manual lesion demarcation in the chronic T1 dataset was comparable to that seen in the acute datasets.

Taken together, compared to the (gold) standard of manual lesion demarcation, the semi-automated *Clusterize* algorithm is capable of significantly speeding up lesion demarcation without loss of either precision or reproducibility. Moreover, our results demonstrate that *Clusterize* is capable of lesion demarcation that is of comparable quality to that obtained by manual lesion demarcation in both acute and chronic stroke and with images in the most commonly used modalities (CT, DWI, T2FLAIR and T1). Finally, the semi-automated approach taken here allows for a routine human quality control step not implemented in fully-automated methods, avoiding the perpetuation of lesion classification errors into later analyses. As this algorithm has been integrated into a simple, freely available SPM toolbox (http://www.medizin.uni-tuebingen.de/kinder/en/research/neuroimaging/software/), it can be effortlessly implemented and used to aid in the preparation of imaging data for larger-scale lesion–behaviour mapping analyses.

## Figures and Tables

**Table 1:** Patient characteristics for each of the 3 acute datasets.

Dataset	Gender	Mean age	Time between stroke onset and imaging	Lesion side	Lesion location
CT	6 M, 7 F	65.9 years (42–80)	2.4 days (0–10)	4 LH, 9 RH	12 MCA, 1 MCA/PCA
DWI	10 M, 6 F	58.4 years (42–80)	2.6 days (0–9)	1 LH, 15 RH	15 MCA, 1 PCA
T2FLAIR	12 M, 3 F	57.8 years (41–80)	3.9 days (0–8)	1 LH, 14 RH	13 MCA, 2 PCA

Legend: for age (at the time of imaging) and time between stroke onset and imaging the mean and range (in brackets) is given. For lesion side, LH = left hemisphere and RH = right hemisphere. For lesion location, MCA = medial cerebral artery territory and PCA = posterior cerebral artery territory.
